# Improvement of La_0.8_Sr_0.2_MnO_3−δ_ Cathode Material for Solid Oxide Fuel Cells by Addition of YFe_0.5_Co_0.5_O_3_

**DOI:** 10.3390/ma15020642

**Published:** 2022-01-15

**Authors:** Michał Mosiałek, Małgorzata Zimowska, Dzmitry Kharytonau, Anna Komenda, Miłosz Górski, Marcel Krzan

**Affiliations:** 1 Jerzy Haber Institute of Catalysis and Surface Chemistry, Polish Academy of Sciences, Niezapominajek 8, 30-239 Cracow, Poland; malgorzata.zimowska@ikifp.edu.pl (M.Z.); Dmitry.Kharitonov@ikifp.edu.pl (D.K.); ania951.1999@gmail.com (A.K.); milosz.gorsky@gmail.com (M.G.); marcel.krzan@ikifp.edu.pl (M.K.); 2 Faculty of Materials Science and Ceramics, AGH University of Science and Technology, Al. Mickiewicza 30, 30-059 Cracow, Poland; 3 Research and Development Center of Technology for Industry, Ludwika Warynskiego 3A, 00-645 Warsaw, Poland

**Keywords:** solid oxide fuel cell, yttrium iron cobaltite, lanthanum strontium manganite, composite cathode, electrochemical impedance spectroscopy

## Abstract

The high efficiency of solid oxide fuel cells with La_0.8_Sr_0.2_MnO_3−δ_ (LSM) cathodes working in the range of 800–1000 °C, rapidly decreases below 800 °C. The goal of this study is to improve the properties of LSM cathodes working in the range of 500–800 °C by the addition of YFe_0.5_Co_0.5_O_3_ (YFC). Monophasic YFC is synthesized and sintered at 950 °C. Composite cathodes are prepared on Ce_0.8_Sm_0.2_O_1.9_ electrolyte disks using pastes containing YFC and LSM powders mixed in 0:1, 1:19, and 1:1 weight ratios denoted LSM, LSM1, and LSM1, respectively. X-ray diffraction patterns of tested composites reveal the presence of pure perovskite phases in samples sintered at 950 °C and the presence of Sr_4_Fe_4_O_11_, YMnO_3_, and La_0.775_Sr_0.225_MnO_3.047_ phases in samples sintered at 1100 °C. Electrochemical impedance spectroscopy reveals that polarization resistance increases from LSM1, by LSM, to LSM2. Differences in polarization resistance increase with decreasing operating temperatures because activation energy rises in the same order and equals to 1.33, 1.34, and 1.58 eV for LSM1, LSM, and LSM2, respectively. The lower polarization resistance of LSM1 electrodes is caused by the lower resistance associated with the charge transfer process.

## 1. Introduction

The slow kinetics of oxygen reduction reaction (ORR) on a cathode is the decisive factor for energy losses in solid oxide fuel cells (SOFCs). La_1−x_Sr_x_MnO_3−δ_ (LSM) was the most frequently used cathode material for SOFC since 1973 [1]. LSM cathodes are effective in ORR at high temperatures (HT) (800–1000 °C) [2]. However, the rapid degradation of cells operating in the HT range prompted researchers to search for cathode materials suitable for SOFCs operating in the 500–800 °C range. In this range, cobalt-containing mixed oxides of the perovskite structure such as La_1−x_Sr_x_CoO_3−δ_, (LSC), Ba_1−x_Sr_x_Co_1−y_Fe_y_O_3−δ_ (BSCF), and La_1−x_Sr_x_Co_1−y_Fe_y_O_3−δ_ (LSCF) reveal superior ionic conductivity compared to LSM. LSCF is an often-used cathode material for comparison in papers reporting new cathode materials working in the IT range. Recently, layered double perovskites are intensively studied due to excellent ORR kinetics [3,4]. Although many new cathode materials have been developed [2,5,6,7,8,9], improvement of physicochemical properties and electrochemical performance of the LSM material remains an urgent task.

LSM reveals excellent mechanical strength, electronic conductivity of 190 S cm^−1^ at 650 °C [10] and 230 S cm^−1^ at 900 °C [11], and a thermal expansion coefficient (TEC) equal to 11.1 × 10^−6^ K^−1^ [6] in the range 300–1270 K, which makes it compatible with frequently used electrolyte materials [12]. These are the main reasons that cause LSM to be more attractive than compared to any other cathode material. It allows quick warm ups and cool downs the cell without degrading the cathode material, providing high operation stability and low susceptibility to failures caused by thermal shocks. Unlike almost all other cathode materials, there is no need to constantly keep LSM-based cells in the operating temperature at off-peak hours, saving its working resource and decreasing operational cost. LSM in reversible solid oxide cells reveals redox instability above 850 °C, whereas below 800 °C it is stable in both oxidizing and reducing atmospheres [13]. The main drawbacks of LSM are its insufficient ionic conductivity in oxidizing atmospheres [11] and a decrease in efficiency in lower temperatures [14]. The performance of LSM cathodes can be improved by an admixture of other cation(s) or by preparing composite electrodes with the addition of a second active phase. Yang et al. [15] prepared LSM-based cathodes in which La was partially replaced by Sr, Nd, Pr, and Sm and demonstrated its stability at 700 °C during 100-hour tests.

The composite cathode material based on LSM can show a synergy effect because individual components of the composite have different advantages resulting in better electrochemical properties than compared to pure LSM. Several composite cathodes containing LSM were examined so far [14,16,17,18,19,20,21,22,23,24,25,26,27,28,29,30,31,32,33,34,35,36,37,38]. The second phase was reported: (i) the electrolyte material of pure ionic conductivity such as yttria-stabilized zirconia (YSZ) [16,17,18,19,20,21], scandia-stabilized zirconia [22], gadolinia-doped ceria (GDC) [18,23] samaria-doped ceria (SDC) [24], bismuth oxide conductors [25,26,27], and lanthanum tungstate [28]; (ii) the noble metal of high catalytic activity such as silver [29,30,31] and platinum [28]; (iii) material of mixed ionic electronic conductivity such as LSCF [14,32], La_0.6_Sr_0.4_FeO_3−δ_, (LSF) [31], La_2_Ni_0.5_Co_0.5_O_4_, and LaNi_0.5_Co_0.5_O_3_ [33]; others such as BaO [34], CeO_2_ [31], CoO_x_ [35], Co_3_O_4_ [36,37], and FeO_x_ [38].

The improvement in the electrochemical performance of LSM with YSZ addition is caused by spatial enlargement of the triple-phase boundary area, increasing the number of electrochemically active sites for charge transfer reaction and oxygen adsorption [16]. Lee et al. [17] found that dissociative adsorption is the main rate-determining step of the ORR of LSM–YSZ composites and found that the best performances exhibit cathodes sintered at 1100 °C. Murray and Barnett [23] described LSM–GDC composite electrodes with a low-current interfacial resistance two to three times lower than LSM–YSZ composite ones. Other doped cerias such as Ce_0.8_Ln_0.2_O_2−δ_ (Ln = Gd, Er, Tb and Pr) and CoO_x_ were added to LSM by Navarrete et al. [35]. The authors found that Pr Ce_0.8_Pr_0.2_O_2−δ_ exhibits the best electrochemical performance. Pajot et al. [26] optimized the strontium content in LSM/Bi_1.5_Er_0.5_O_3_-composite electrodes. These authors showed that these cathodes have *R*_p_ of 0.1 and 3 Ω cm^2^ at 675 and 500 °C, respectively. Strandbakke et al. [28] reported that composite cathodes LSM with lanthanum tungstate and platinum reveal polarization resistance 40 Ω cm^2^ at 650 °C. Rehman et al. [18] described the properties of ternary composite LSM–YSZ–GDC, reporting maximum power densities of 215, 316, and 396 mW cm^−2^ at 750, 800, and 850 °C, respectively. Wang et al. [24] applied LSM–SDC composite cathode in low-temperature SOFC. The authors reported that the optimum ratio of LSM:SDC is 1:2. LT-SOFC with this material reached a power density of 742 mW cm^−2^ under 550 °C. The enhanced catalytic activities of LSM cathode-infiltrated metallic (Ag), ceramic LSF and CeO_2_ nanocatalysts were reported by Seyed-Vakili et al. [31]. The authors found that the co-infiltration of Ag and ceria reduced the polarization resistance of the electrode to 2.5% of that for the pure LSM electrode. Furthermore, the infiltration of Ag solution reduced electrode overpotential by about 114% at 700 °C. Li et al. [36] assembled Co_3_O_4_ catalysts on the LSM cathode’s surface by using a hydrothermal method. The polarization resistance of the symmetric cell with the LSM cathode and modified LSM cathode was 0.95 Ω cm^2^ and 0.55 Ω cm^2^ at 700 °C, respectively. Shahrokhi et al. [33] infiltrated LSM oxygen electrodes with nanoparticles of La_2_Ni_0.5_Co_0.5_O_4_ of the Ruddlesden-Popper structure and LaNi_0.5_Co_0.5_O_3_ of the perovskite structure and found that La_2_Ni_0.5_Co_0.5_O_4_ has greater impact than LaNi_0.5_Co_0.5_O_3_ on both the decrease in polarization resistance and stability of the electrode under cyclic solid oxide electrolysis cell (SOEC) and SOFC modes. Liu et al. [14] tested LSM–LSCF composite cathodes. The authors found that a cell with an LSM-infiltrated LSCF cathode revealed at 825 °C a peak power density of 1.07 W cm^−2^, about 24% higher than that of the same cell without LSM infiltration. LSM was studied as an electrode material for SOFC operating reversibly as SOECs [2,39], which are promising for the storage and regeneration of sustainable energy from solar and wind power energy sources. Zheng et al. [40] studied La_0.8_Sr_0.2_Co_0.8_Ni_0.2_O_3−δ_-impregnated LSM–GDC composites, including oxygen electrodes. The authors reported a peak power density of 1057 mW cm^−2^ at 800 °C in the SOFC mode. In co-electrolysis modes, the current density reached 1.60 A cm^−2^ at 1.5 V at 800 °C with an H_2_O/CO_2_ ratio of 2/1. Yu et al. [38] infiltrated FeO_x_ nanoparticles onto an LSM backbone cathode. The infiltration process resulted in an increase of 3.4% in cell performance. LSM is also frequently used as a current collector layer [41] or a protective layer on a stainless-steel current collector.

Currently, mixed oxides of perovskite structure of the general formula ABO_3_ are widely used in SOFC applications. Among them, YFe_0.5_Co_0.5_O_3_ (YFC) reveals a polarization resistance of 0.07 Ω cm^2^ at 750 °C, TEC of 17.5 × 10^−6^ K^−1^, and conductivity of 183 S cm^−1^ [42]. The excellent properties of YFC as a cathode material make it a promising candidate for implementation in the composite cathode for SOFCs. However, according to our knowledge, no other attempt has been made to develop the YFC-containing composite cathodes for SOFC application.

Porosity performs an essential function in enhancing the performance of SOFC electrodes because gas-phase diffusion resistance can be significant, even far away from the limiting current [43]. Controlling porosity can improve electrode performance by facilitating gas diffusion [44]. Laguna-Bercero et al. [45] reported microbeads with 6 μm diameter is a good pore former for generating a microstructure that works well in both SOEC and SOFC modes.

This study aims to improve the properties of LSM cathodes working in the range of 500–800 °C by the adding YFe_0.5_Co_0.5_O_3_. In order to precisely determine which parameters of the electrode process are affected by a given additive, all tests were carried out by using LSM without the addition of electrolyte materials (YSZ and GDC). The current collector layer has a huge impact on cathode efficiency [46,47]; therefore, such a layer was not used in the described experiments.

## 2. Materials and Methods

### 2.1. Synthesis of YFC Powder

YFC was synthesized by using the citric acid method. At first, Y_2_O_3_ (supplied by Onyxmet, Olsztyn, Poland) was dissolved in nitric acid (Sigma-Aldrich, Saint Louis, USA, reagent grade) to obtain a transparent solution containing yttrium cations. Then, cobalt and iron nitrates (supplied by Sigma-Aldrich, Saint Louis, USA, reagent grade) in stoichiometric amounts (Y:Fe:Co = 1:0.5:0.5) were added. The obtained solution was continuously stirred with added dropwise citric acid under heating at 80 °C until the gel was obtained. The gel was dried at 110 °C to obtain bottle-green xerogel and then sintered at 950 °C for 10 h or 1100 °C for 10 h, as described in more detail in [42].

### 2.2. Characterization

In order to identify the crystalline phases formed after sintering LSM/YFC mixtures at different temperatures, X-ray diffraction patterns were collected in the 2θ range of 2–72° with the use of an X’Pert PRO (PANalytical B.V., Cambridge, UK) diffractometer operated at 40 kV and 30 mA by applying Ni-filtered Cu K_α_ radiation. Before measurements, the samples were uploaded on a silicon low-background sample holder. A Field Emission Scanning Electron Microscope JEOL JSM-7500F (JEOL Ltd. Tokyo, Japan) equipped with the RBEI retractable backscattered electron detector and INCA PentaFetx3 EDS detection system of characteristic X-ray radiation [48] was applied for imaging the morphology of the obtained composite cathodes.

The porosity and BET-specific surface area of the samples were determined at 196 °C from nitrogen adsorption-desorption isotherms with the use of a Quantachrome Nova 2000 apparatus (Quantachrome Instruments, Boynton Beach, FL, USA) after outgassing for 3 h at 200 °C under vacuum.

### 2.3. Preparation of Cells

In order to prepare cathode materials, synthesized YFC and La_0.8_Sr_0.2_MnO_3−δ_ (LSM) (received from Nexceris, OH, USA) powders were mixed in assumed weight ratios, 0:100, 5:95, and 50:50, with an ink vehicle (Nexceris, OH, USA), and Spheromers CA6 polystyrene balls of 6 μm in diameter (Microbeads AS, Skedsmokorset, Norway) were used as pore formers until homogeneous ink was achieved. The obtained compositions are denoted as LSM, LSM1, and LSM2, respectively.

**Figure 1:**
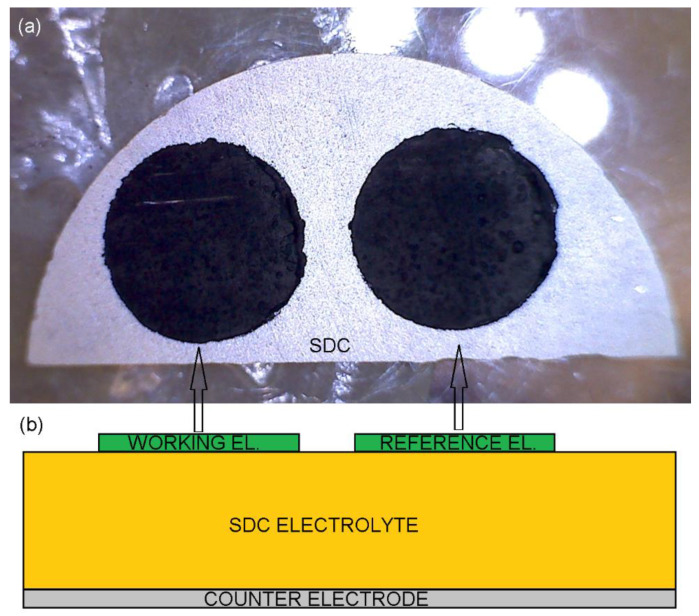
The sintered cell (**a**) top view photography and (**b**) side-view sketch.

The round working and reference electrodes of 5 mm in diameter were printed on 1-millimeter-thick SDC electrolyte half disks of 20 mm in diameter, prepared as described in [49] and covered by LSCF at the opposite side [49]. Then, cells were sintered at 950 °C or 1100 °C for 2 h (Figure 1).

### 2.4. The Electrochemical Properties

The electrochemical properties of all electrodes were tested by using a Gamry 300 series potentiostat/galvanostat/ZRA (Gamry Instruments, Warminster, PA, USA) in gas mixtures of set oxygen partial pressures from 0.001 to 1 containing oxygen (99.5%, Air Liquide Polska Sp. z o. o., Kraków, Polska) or oxygen and argon (99.999%, Air Liquide Polska Sp. z o. o., Kraków, Polska) with a gas flow rate of 150 cm^3^ min^−1^. The cell was mounted in the alumina test fixture for electrochemical measurements as reported elsewhere [50]. Electrochemical impedance spectroscopy (EIS) spectra were recorded in a frequency range from 8 mHz to 300 kHz with a density of 8 frequency points per decade with AC voltage signal of 5 or 20 mV rms. Data treatment based on the program MINUIT [51] is described in detail elsewhere [50].

## 3. Results and Discussion

**Figure 2:**
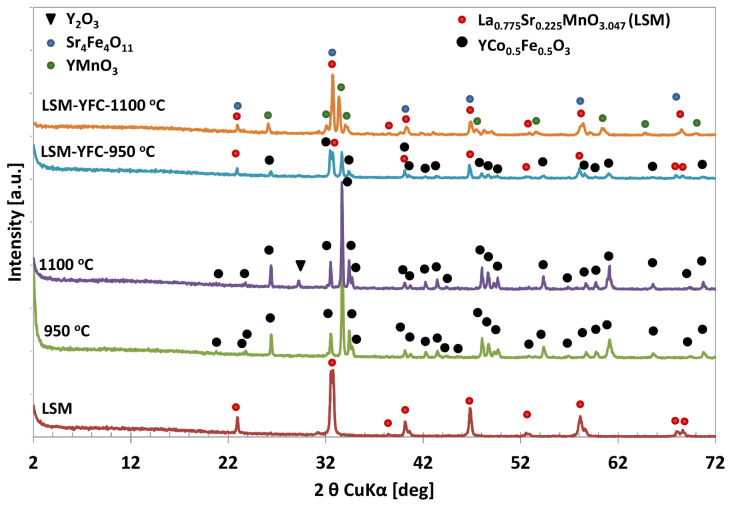
XRD patterns of YFC sintered at 950 °C (**green**) and 1100 °C (**violet**); LSM (**brown**); YFC mixed with LSM and sintered at 950 °C (**blue**) and 1100 °C (**orange**).

The assessment of thermal transformation of pure YFC xerogel and its co-mixture with LSM was analyzed by the XRD powder diffraction method. Calcination of the as-synthesized, bottle-green xerogel at 950 °C resulted in the formation of a highly crystalline YFe_0.5_Co_0.5_O_3_ perovskite structure with orthorhombic Pnma symmetry [52], whereas xerogel sintered at 1100 °C additionally contained a small amount of Y_2_O_3_ (Figure 2) pointing at the partial decomposition of YFC. A thorough analysis of the XRD pattern (Figure 2) indicated a slight difference in phase composition of LSM/YFC mixture as a function of calcination temperature. The diffraction pattern of the LSM and YFC mixture sintered at 950 °C for 2 h showed reflections belonging to La_0.775_Sr_0.225_MnO_3.047_ [53] and YFe_0.5_Co_0.5_O_3_ [52] phases. Sintering the LSM/YFC mixture at 1100 °C for two hours resulted in the appearance of new oxide phases due to chemical reactions in the solid state.

Analysis of the XRD pattern (Figure 2) indicated that the LSM/YFC mixture sintered at 1100 °C is composed of a mixture of crystalline Sr_4_Fe_4_O_11_, YMnO_3_, and La_0.775_Sr_0.225_MnO_3.047_ oxide phases. The presence of the Sr_4_Fe_4_O_11_ phase is uncertain due to its reflections appearing at the same 2θ values as reflections of the LSM phase. However, the increase in the intensity of reflection at 32.70° of 2θ may confirm its contribution.

**Figure 3:**
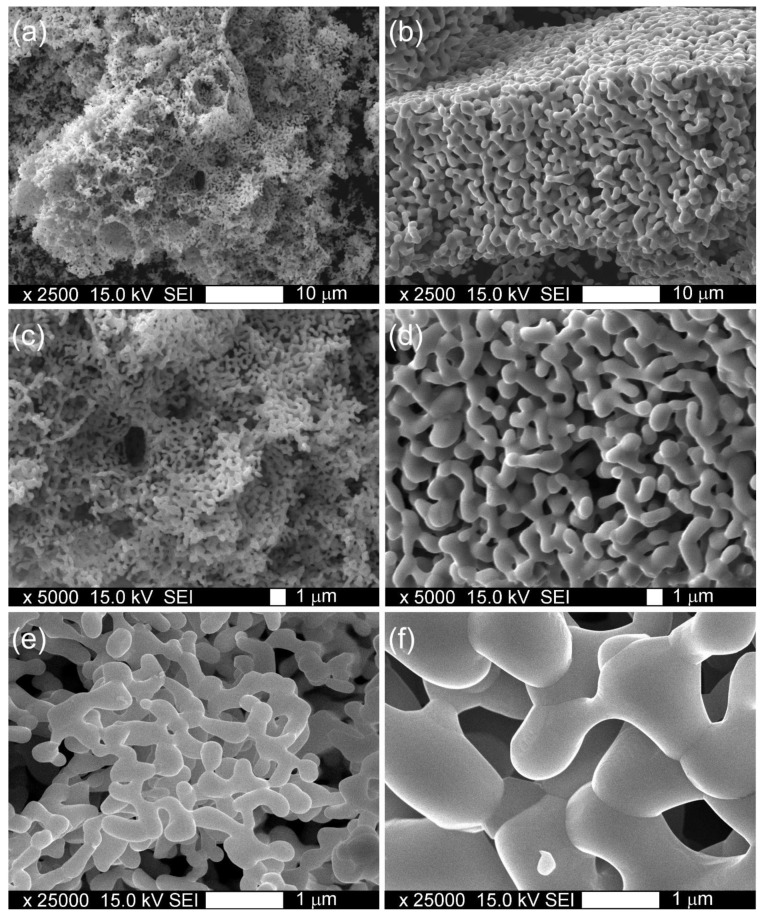
Morphology of YFC powders sintered at 950 °C (**a**,**c**,**e**) and 1100 °C (**b**,**d**,**f**).

The morphology of obtained YFC perovskite powders is presented in Figure 3. The highly crystallized perovskite structure obtained after calcination at 950 °C consists of small, several millimeter-long fragile wafer-like pieces and revealed a specific surface area of 6 m^2^ g^−1^. The increase in sintering temperature up to 1100 °C caused a merging of small structures into larger ones accompanied by a decrease in BET surface area to 4 m^2^ g^−1^.

**Figure 4:**
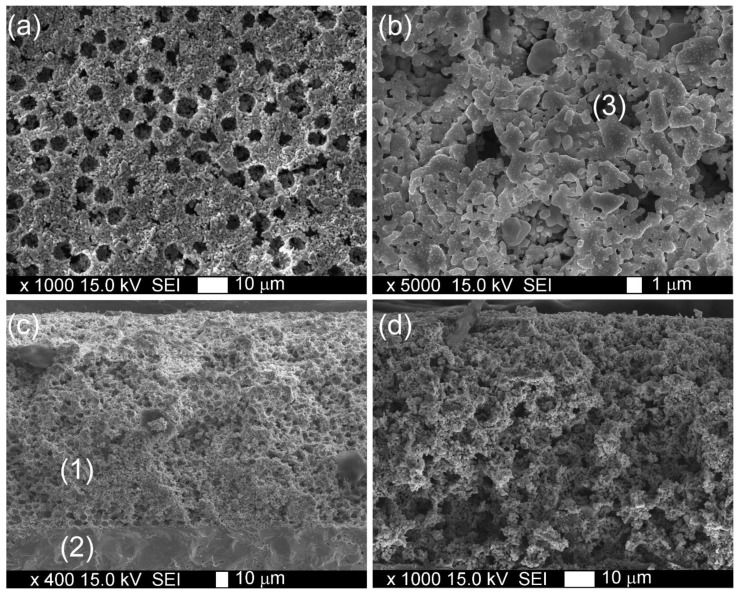
SEM images: (**a**,**b**) surface and (**c**,**d**) cross section of LSM1 electrode sintered at 1100 °C; (1) cathode, (2) electrolyte, and (3) pore formed by microbead.

The morphology of the formed LSM1 composite cathode sintered at 1100 °C is presented in Figure 4. SEM images and point EDS analysis revealed a homogenous distribution of Y, Fe, and Co in the LSM matrix. Numerous pores of ~6 µm in diameter originating from the thermal removal of polystyrene templates are observed in the microstructure of the formed cathode. Such a uniform distribution of pores in the microstructure facilitates oxygen transport in the electrode and reduces energy losses.

**Figure 5:**
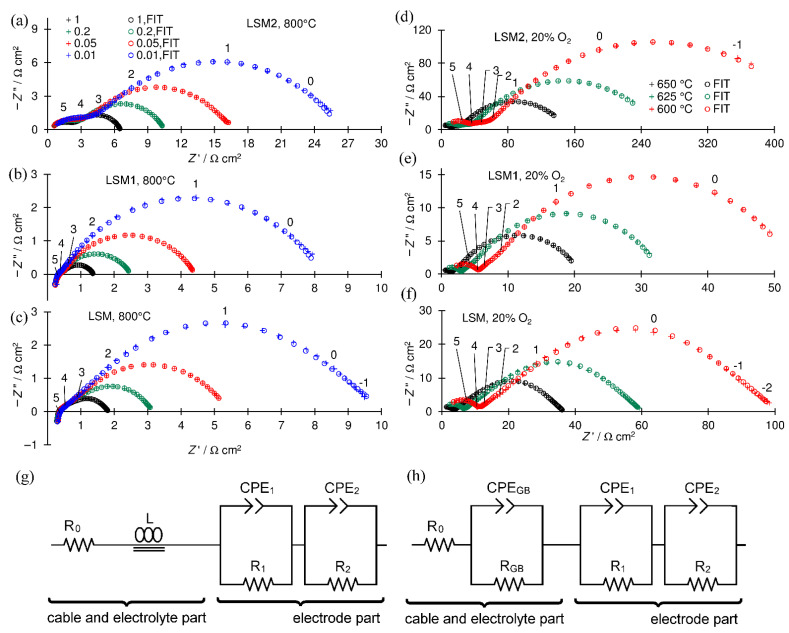
Example of recorded EIS spectra: (**a**–**c**) t 800 °C and *P*(O_2_) *P*^−1^ = 1, 0.2, 0.05, and 0.01 for black, green, red, and blue, respectively; (**d**–**f**) at *P*(O_2_) *P*^−1^ = 0.2, and at 650, 625, and 600 °C— for black, green, and red, respectively; (**a**,**d**) LSM2 cathode; (**b**,**e**) LSM1 cathode; (**c**,**f**) LSM cathode; crosses experimental points, circles fitted points, numbers above enlarged crosses denote the logarithm of the frequency, and (**g**,**h**) electrical equivalent circuits used for fitting spectra; all spectra are shifted along the x-axis near the origin to facilitate comparisons.

Examples of recorded EIS spectra are presented in Figure 5. In the majority of EIS spectra recorded at higher temperatures (above 725 °C), the inductive loop caused by cable inductance appears (Figure 5b,c), whereas in spectra recorded at lower temperatures, a part of high-frequency (HF) capacitive semicircle connected to the electrolyte grain boundary resistance is visible (Figure 5d–f). The electrode part of impedance spectra consists of one (Figure 5b,c) or two (Figure 5a,d–f) overlapped capacitive semicircles. According to Wang et al. [54], the LSM electrode spectra can be divided into up to three semicircles representing electrode processes. The HF semicircle represents the charge transfer process, the medium frequency (MF) semicircle, the oxygen ion transfer on the electrode–electrolyte interface, and the low frequency (LF) semicircle surface-exchange reaction on the cathode’s surface consisting of dissociative adsorption of oxygen, ionization of adsorbed oxygen atoms to O^2−^ ions, and incorporation of these ions into the LSM electrode. The other authors [17] ascribed the LF process to gas diffusion according to the Adler–Lane–Steele Model [43].

The obtained spectra were analyzed by the complex nonlinear least-squares method. Used electrical equivalent circuits (EECs) presented in Figure 5g,h are divided into two parts connected in series. The first electrolyte part contains resistor R_0_, which represents the resistance of lead cables and the part of the electrolyte resistance that is not visible in the frequency range of the recorded spectrum, whole or the grain interior electrolyte resistance, and coil L (Figure 5e) concerning the spectra in which the inductive loop representing the inductance of the cables is present. Each capacitive semicircle in the spectra is represented in EEC by a subcircuit consisting of a parallel-connected resistor (R) and a constant phase element (CPE).

The semicircle connected with the impedance of the grain boundary of the SDC electrolyte resistance observed in a part of the spectra is represented by the (R_GB_ and CPE_GB_) subcircuit. The electrode part of the EEC consists of two (R_1_ and CPE_1_) and (R_2_ and CPE_2_) or one (R_1_ and CPE_1_) subcircuit for spectra with two (Figure 5a,d–f) or one (Figure 5b,c) semicircle in the electrode parts, respectively. The polarization resistance *R*_p_ was calculated as the sum of resistances representing electrode processes *R*_p_ = *R*_1_ + *R*_2_ for two or *R*_p_ = *R*_1_ for one (R and CPE) subcircuit in the electrode part of the EEC.

LSM does not react with YFC at 950 °C; however, it sinters very poorly at this temperature. The cathodes obtained at 950 °C were characterized by a very high *R*_p_; thus, the results presented below concern only the cathodes obtained at 1100 °C.

**Figure 6:**
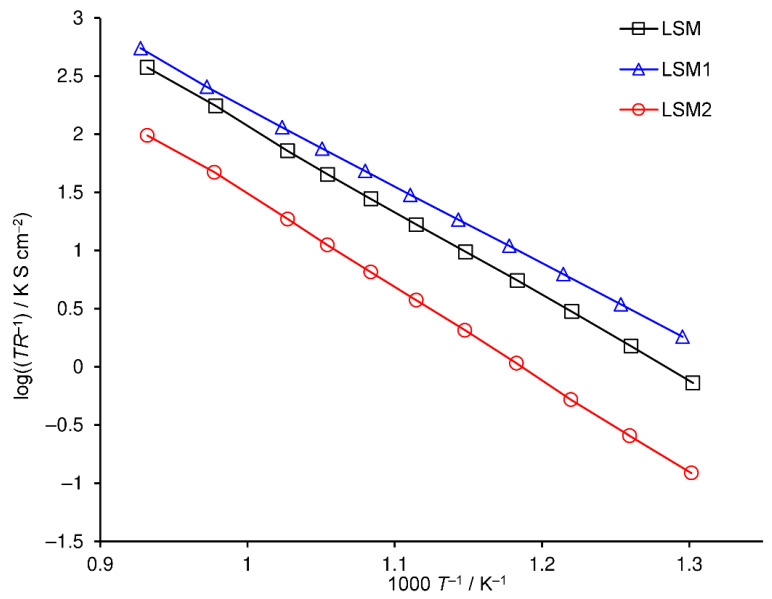
Arrhenius plot of reciprocal polarization resistance of cathodes sintered at 1100 °C, LSM (black squares), LSM1 (blue triangles), and LSM2 (red circles).

The dependency of polarization resistance on temperature is presented in the Arrhenius plot (Figure 6). LSM1 and LSM2 cathodes attain polarization resistances lower and greater than the LSM one, respectively, in the entire examined temperature range. Moreover, the differences between the polarization resistances of LSM, LSM1 and LSM2 electrodes increase with decreasing temperature due to differences in the values of its activation energy (*E*_a_). Activation energies of reciprocal polarization resistances in the range of 500–800 °C are equal to 1.34, 1.33, and 1.58 eV for LSM, LSM1, and LSM2 cathodes, respectively. Huber et al. [55] reported an *E*_a_ of 1.4 eV for LSM in the range below 700 °C.

In order to identify the influence of YFC addition on different rate-limiting steps of the ORR, resistance dependency associated with each response was studied as a function of *P*(O_2_). The dependence of the resistance associated with the *i*-th process on oxygen concentration is described by the following formula:

log(*R*_i_) = *a* − *m* log(*P*(O_2_)), (1)

where *a* and *m* are coefficients. The *m* value is the reaction order and provides information about oxygen species involved in this stage of the electrode reaction [17,56,57,58,59,60].

*m* = 0 oxygen ion transfer on the electrode–electrolyte interface.

*m* = 0.25, the charge transfer process.

(2) $$O_{\mathrm{ads}}+2e^{-}+V_{O}^{..}=O_{O}^{x}$$

*m* = 0.375, the one electron charge transfer process.

(3) $$O_{\mathrm{ads}}+e^{-}=O_{\mathrm{ads}}^{-}$$

*m* = 0.5, dissociation of adsorbed oxygen molecule.

(4) $$O_{2,\mathrm{ads}}=2O_{\mathrm{ad}}^{\;}$$

**Figure 7:**
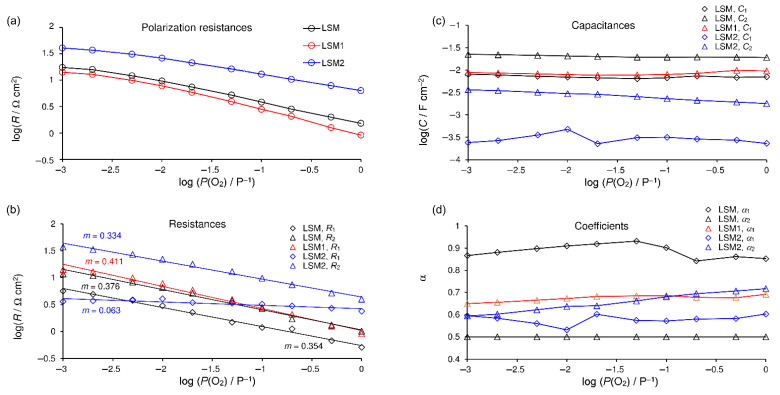
Relationships for spectra recorded at 800 °C on oxygen partial pressure: (**a**) polarization resistance and results of fitting for separate processes: (**b**)—resistances with trend lines and calculated *m* coefficients, (**c**) capacitances, and (**d**) parameters *α*. LSM electrode—black; LSM1—red; LSM2—blue.

The dependencies of the fitted parameters on the oxygen pressure at 800 °C are shown in Figure 7. The EECs containing either three or two (R, CPE) subcircuits were tested for fitting EIS spectra of the LSM electrode. Results were characterized by standard deviation in the range from 0.11 to 0.98% and 0.62–2.03%, respectively. Therefore, the EEC containing three (R, CPE) subcircuits was chosen for the LSM cathode. Figure 7b–d show the values of the fitted parameters. C_GB_, R_GB_, and α_GB_ are constant across the entire concentration range, and their values are typical for GB SDC electrolyte impedance [49] for all spectra where GB semicircle appeared. *R*_1_ and *R*_2_ are oxygen concentration dependent and characterized by reaction orders close to 0.375, which is characteristic for the one-electron charge-transfer process (Figure 7b). Both processes are characterized by an identical dependence on oxygen pressure within the measurement error and small differences in capacity, however, coefficients *α* are very different, e.g., around 0.5 and 0.9, causing the difference in semicircle shapes. Therefore, it should not be treated as a single process. Yan et al. [61] observed that surface exchange reaction depends on the crystallographic surface on which it occurs. These authors proposed two parallel surface exchange mechanisms.

The EEC containing two (R, CPE) subcircuits was used for fitting EIS spectra of the LSM1 electrode. The results were characterized by a standard deviation in the range from 0.18 to 0.42. *R*_1_ is oxygen-concentration dependent and characterized by reaction order *m* = 0.41, which is higher than for the pure LSM cathode. The slightly distorted capacitive semicircle assigned to the electrode reaction consists of two overlapped semicircles representing processes of different reaction orders 0.375 and 0.5. The increase in the order of the reaction caused by small YFC additions means that the resistance caused by dissociation of adsorbed oxygen molecules has a greater share. Since the entire resistance decreased simultaneously, we conclude that it is caused by a decrease in resistance associated with the one-electron charge-transfer process.

The EEC containing two (R, CPE) subcircuits in its electrode part is used for fitting EIS spectra of the LSM2 electrode. The results were characterized by a standard deviation in the range from 0.29 to 0.66%. *R*_1_ is oxygen-concentration dependent and characterized by reaction order *m* = 0.33 (*m* = 0.39 in the range *P*(O_2_) *P*^−1^ from 0.1 to 1); *R*_2_ is almost oxygen-concentration independent with *m* = 0.06, which confirms that the MF process constitutes oxygen ion transfer on the electrode–electrolyte interface (*m* = 0 [60]).

The EEC containing two (R, CPE) subcircuits was used for fitting EIS spectra for LSM and LSM1 electrodes at 700 °C as well as three (R, CPE) subcircuits for LSM2. The orders of reactions at this temperature are similar to those obtained at 800 °C. For LSM and LSM1 cathodes, *m* = 0.41 and *m* = 0.43 were obtained, respectively, whereas for LSM2, *R*_1_ is characterized by reaction order *m* = 0.36 (*m* = 0.43 in the range *P*(O_2_) *P*^−1^ from 0.1 to 1) and *m* = 0.08 for *R*_2_.

## 4. Conclusions

In this study, highly porous composite cathodes made of LSM and YFC for IT SOFCs were obtained for the first time. The following conclusions are made:

1. The sintering of YFC xerogel at 950 °C results in the formation of a pure, highly crystalline, wafer-like perovskite structure. Composite cathodes sintered at 950 °C LSM1 (5 wt% YFC—95 wt% LSM) and LSM2 (50 wt% YFC—50 wt% LSM) maintained both components’ structure; however, they were characterized by very high polarization resistance.
2. The increase in sintering temperature up to 1100 °C causes the disintegration of perovskite crystals and the creation of Sr_4_Fe_4_O_11_ and YMnO_3_ mixed oxide phases. These cathodes reveal acceptably low polarization resistance and activation energies equal to 1.34, 1.33, and 1.58 eV for LSM, LSM1, and LSM2 respectively.
3. The 5 wt% addition of YFC to LSM cathodes results in a decrease in polarization resistance. The decrease is probably caused by the facilitation of the charge transfer process. The decrease becomes larger with a decrease in temperature and with an increase in oxygen concentration.

## Data Availability

The data presented in this study are available upon request from the corresponding authors.

## References

[1] Adler S.B. (2004). Factors governing oxygen reduction in solid oxide fuel cell cathodes. Chem. Rev..

[2] Mogensen M.B. (2020). Materials for reversible solid oxide cells. Curr. Opin. Electrochem..

[3] Pelosato R., Cordaro G., Stucchi D., Cristiani C., Dotelli G. (2015). Cobalt based layered perovskites as cathode material for intermediate temperature Solid Oxide Fuel Cells: A brief review. J. Power Sources.

[4] Volkova N.E., Mychinko M.Y., Golovachev I.B., Makarova A.E., Bazueva M.V., Zyaikin E.I., Gavrilova L.Y., Cherepanov V.A. (2019). Structure and properties of layered perovskites Ba_1-x_Ln_x_Fe_1-y_Co_y_O_3−δ_ (Ln = Pr, Sm, Gd). J. Alloys Compd..

[5] Tsipis E.V., Kharton V.V. (2008). Electrode materials and reaction mechanisms in solid oxide fuel cells: A brief review: I Performance-determining factors. J. Solid State Electrochem..

[6] Tsipis E.V., Kharton V.V. (2008). Electrode materials and reaction mechanisms in solid oxide fuel cells: A brief review. II. Electrochemical behavior vs. materials science aspects. J. Solid State Electrochem..

[7] Tsipis E.V., Kharton V.V. (2011). Electrode materials and reaction mechanisms in solid oxide fuel cells: A brief review. III. Recent trends and selected methodological aspects. J. Solid State Electrochem..

[8] Mahato N., Banerjee A., Gupta A., Omar S., Balani K. (2015). Progress in Materials Science Progress in material selection for solid oxide fuel cell technology: A review. Prog. Mater. Sci..

[9] Kaur P., Singh K. (2020). Review of perovskite-structure related cathode materials for solid oxide fuel cells. Ceram. Int..

[10] Gupta R.K., Kim E.Y., Kim Y.H., Whang C.M. (2009). Thermal, micro-structural, and electrical properties of a La_1-x_Sr_x_Mn0_.85_Fe_0.05_Co_0.05_Ni_0.05_O_3+δ_ (x = 0–0.4 mole) cathode system. Met. Mater. Int..

[11] Corbel G., Mestiri S., Lacorre P. (2005). Physicochemical compatibility of CGO fluorite, LSM and LSCF perovskite electrode materials with La_2_Mo_2_O_9_ fast oxide-ion conductor. Solid State Sci..

[12] Kharton V.V., Marques F.M.B., Atkinson A. (2004). Transport properties of solid oxide electrolyte ceramics: A brief review. Solid State Ion..

[13] Luo X., Yang Y., Yang Y., Tian D., Lu X., Chen Y., Huang Q., Lin B. (2018). Reduced-temperature redox-stable LSM as a novel symmetrical electrode material for SOFCs. Electrochim. Acta.

[14] Liu Z., Liu M. (2013). LSM-infiltrated LSCF cathodes for solid oxide fuel cells. J. Energy Chem..

[15] Yang Y., Bao H., Ni H., Ou X., Wang S., Lin B., Feng P., Ling Y. (2021). A novel facile strategy to suppress Sr segregation for high-entropy stabilized La_0.8_Sr_0.2_MnO_3−δ_ cathode. J. Power Sources.

[16] Wang S., Jiang Y., Zhang Y., Yan J., Li W. (1998). Promoting effect of YSZ on the electrochemical performance of YSZ + LSM composite electrodes. Solid State Ion..

[17] Lee E., Jeong H., Shin T.H., Myung J.H. (2021). Determination of the rate-determining step of the oxygen reduction reaction of La_0.8_Sr_0.2_MnO_3_(LSM)- 8mol% yttria-stabilized zirconia(YSZ): Composition and microstructure. Ceram. Int..

[18] Rehman S.U., Song R.H., Lee J.W., Lim T.H., Park S.J., Lee S.B. (2016). Effect of GDC addition method on the properties of LSM–YSZ composite cathode support for solid oxide fuel cells. Ceram. Int..

[19] Nielsen J., Hjelm J. (2014). Impedance of SOFC electrodes: A review and a comprehensive case study on the impedance of LSM:YSZ cathodes. Electrochim. Acta.

[20] Jung G.B., Chang C.T., Yeh C.C., Nguyen X.V., Chan S.H., Lin C.Y., Yu J.W., Lee W.T., Chang S.W., Kao I.C. (2016). Study of reversible solid oxide fuel cell with different oxygen electrode materials. Int. J. Hydrog. Energy.

[21] Agbede O.O., Hellgardt K., Kelsall G.H. (2020). Electrical conductivities and microstructures of LSM, LSM-YSZ and LSM-YSZ/LSM cathodes fabricated on YSZ electrolyte hollow fibres by dip-coating. Mater. Today Chem..

[22] Nagasawa T., Hanamura K. (2017). Particle-Scaled Visualization of Active Sites in LSM/ScSZ Composite Cathode of SOFC through Oxygen Isotope Labeling. ECS Trans..

[23] Murray E.P., Barnett S.A. (2001). (La,Sr)MnO_3_–(Ce,Gd)O_2–x_ composite cathodes for solid oxide fuel cells. Solid State Ion..

[24] Wang Z., Wang X., Xu Z., Deng H., Dong W., Wang B., Feng C., Liu X., Wang H. (2018). Semiconductor-ionic nanocomposite La_0.1_Sr_0.9_MnO_3−δ_−Ce_0.8_Sm_0.2_O_2−δ_ functional layer for high performance low temperature SOFC. Materials.

[25] Kim S.J., Dayaghi A.M., Kim K.J., Choi G.M. (2017). Er_0.4_Bi_1.6_O_3−δ_—La_0.8_Sr_0.2_MnO_3−δ_ nano-composite as a low-temperature firing cathode of solid oxide fuel cell. J. Power Sources.

[26] Pajot M., Duffort V., Capoen E., Mamede A.S., Vannier R.N. (2020). Influence of the strontium content on the performance La_1-x_Sr_x_MnO_3_/Bi_1.5_Er_0.5_O_3_ composite electrodes for low temperature Solid Oxide Fuel Cells. J. Power Sources.

[27] Lee K.T., Jung D.W., Yoon H.S., Camaratta M., Sexson N., Wachsman E.D. (2019). High Performance LSM-ESB Cathode on ESB Electrolyte for Low to Intermediate Temperature Solid Oxide Fuel Cells. ECS Trans..

[28] Strandbakke R., Dyrlie O., Hage F.S., Norby T. (2016). Reaction Kinetics of Protons and Oxide Ions in LSM/Lanthanum Tungstate Cathodes with Pt Nanoparticle Activation. J. Electrochem. Soc..

[29] Sholklapper T.Z., Radmilovic V., Jacobson C.P., Visco S.J., Jonghe L.C. (2008). De Nanocomposite Ag—LSM solid oxide fuel cell electrodes. J. Power Sources.

[30] Mosialek M., Przybyła M., Tatko M., Nowak P., Dudek M., Zimowska M. (2013). Composite Composite Ag-La_0.8_Sr_0.2_MnO_3−δ_ cathode for solid oxide fuel cells. Arch. Metall. Mater..

[31] Seyed-Vakili S.V., Babaei A., Ataie M., Heshmati-Manesh S., Abdizadeh H. (2018). Enhanced performance of La_0.8_Sr_0.2_MnO_3_ cathode for solid oxide fuel cells by co-infiltration of metal and ceramic precursors. J. Alloys Compd..

[32] Ai N., Chen K., Jiang S.P. (2016). A La_0.8_Sr_0.2_MnO_3_/La_0.6_Sr_0.4_Co_0.2_Fe_0.8_O_3−δ_ core–shell structured cathode by a rapid sintering process for solid oxide fuel cells. Int. J. Hydrog. Energy.

[33] Shahrokhi S., Babaei A., Zamani C. (2018). Reversible operation of La_0.8_Sr_0.2_MnO_3_ oxygen electrode infiltrated with Ruddlesden-Popper and perovskite lanthanum nickel cobaltite. Int. J. Hydrog. Energy.

[34] Traulsen M.L., McIntyre M.D., Norrman K., Sanna S., Mogensen M.B., Walker R.A. (2016). Reversible Decomposition of Secondary Phases in BaO Infiltrated LSM Electrodes—Polarization Effects. Adv. Mater. Interfaces.

[35] Navarrete L., Balaguer M., Vert V.B., Serra J.M. (2016). Optimization of SOFC Composite Cathodes Based on LSM and Doped Cerias Ce_0.8_Ln_0.2_O_2−δ_ (Ln = Gd, Er, Tb and Pr). J. Electrochem. Soc..

[36] Li M., Cheng J., Gan Y., Xu C. (2018). In situ construction of Co_3_O_4_ nanoarray catalysts on (La_0.8_Sr_0.2_)_0.95_MnO_3−δ_ cathode for high-efficiency intermediate-temperature solid oxide fuel cells. Ceram. Int..

[37] Xu X., Wang C., Fronzi M., Liu X., Bi L., Zhao X.S. (2019). Modification of a first-generation solid oxide fuel cell cathode with Co_3_O_4_ nanocubes having selectively exposed crystal planes. Mater. Renew. Sustain. Energy.

[38] Yu Y., Ohodnicki P., Abernathy H., Fan Y., Kalapos T., Hackett G. (2018). Characterization of Interaction between Fe-Infiltrates and LSM Backbone in Solid Oxide Fuel Cells. Phys. Status Solidi Appl. Mater. Sci..

[39] Chen K., Liu S.S., Ai N., Koyama M., Jiang S.P. (2015). Why solid oxide cells can be reversibly operated in solid oxide electrolysis cell and fuel cell modes?. Phys. Chem. Chem. Phys..

[40] Zheng H., Tian Y., Zhang L., Chi B., Pu J., Jian L. (2018). La_0.8_Sr_0.2_Co_0.8_Ni_0.2_O_3−δ_ impregnated oxygen electrode for H_2_O/CO_2_ co-electrolysis in solid oxide electrolysis cells. J. Power Sources.

[41] Jain S.L., Nabae Y., Lakeman B.J., Pointon K.D., Irvine J.T.S. (2008). Solid state electrochemistry of direct carbon/air fuel cells. Solid State Ion..

[42] Cui J., Wang J., Fan W., Wan Y., Zhang X., Li G., Wu K., Cheng Y., Zhou J. (2017). Porous YFe_0.5_Co_0.5_O_3_ thin sheets as cathode for intermediate-temperature solid oxide fuel cells. Int. J. Hydrog. Energy.

[43] Adler S.B., Lane J.A., Steele B.C.H. (1996). Electrode Kinetics of Porous Mixed-Conducting Oxygen Electrodes. J. Electrochem. Soc..

[44] Hedayat N., Du Y., Ilkhani H. (2018). Pyrolyzable pore-formers for the porous-electrode formation in solid oxide fuel cells: A review. Ceram. Int..

[45] Laguna-Bercero M.A., Hanifi A.R., Menand L., Sandhu N.K., Anderson N.E., Etsell T.H., Sarkar P. (2018). The effect of pore-former morphology on the electrochemical performance of solid oxide fuel cells under combined fuel cell and electrolysis modes. Electrochim. Acta.

[46] Guo Y., Liu Y., Cai R., Chen D., Ran R., Shao Z. (2012). Electrochemical contribution of silver current collector to electrode on oxygen-ionic conducting electrolyte. Int. J. Hydrog. Energy.

[47] Guo Y., Zhou Y., Chen D., Shi H., Ran R., Shao Z. (2011). Significant impact of the current collection material and method on the performance of Ba_0.5_Sr_0.5_Co_0.8_Fe_0.2_O_3−δ_ electrodes in solid oxide fuel cells. J. Power Sources.

[48] Mosiałek M., Dudek M., Michna A., Tatko M., Kędra A., Zimowska M. (2014). Composite cathode materials Ag-Ba_0.5_Sr_0.5_Co_0.8_Fe_0.2_O_3_ for solid oxide fuel cells. J. Solid State Electrochem..

[49] Mosiałek M., Michna A., Dziubaniuk M., Bielańska E., Kežionis A., Šalkus T., Kazakevičius E., Bożek B., Krawczyk A., Wyrwa J. (2018). Composite cathode material LSCF-Ag for solid oxide fuel cells obtained in one step sintering procedure. Electrochim. Acta.

[50] Mosiałek M., Nowak P., Dudek M., Mordarski G. (2014). Electrochimica Acta electrochemical impedance spectroscopy and cyclic voltammetry at the silver point electrode. Electrochim. Acta.

[51] James F., Roos M. (1975). Minuit—A system for function minimization and analysis of the parameter errors and correlations. Comput. Phys. Commun..

[52] Soorya K. (2015). ICDD PDF-4+ 2015 01-084-8433. Database.

[53] Soorya K. (2015). ICDD PDF-4+ 2015 04-014-2109 Database.

[54] Wang W., Jiang S.P. (2006). A mechanistic study on the activation process of (La, Sr)MnO_3_ electrodes of solid oxide fuel cells. Solid State Ion..

[55] Huber T.M., Kubicek M., Opitz A.K., Fleig J. (2015). The Relevance of Different Oxygen Reduction Pathways of La_0.8_Sr_0.2_MnO_3_ (LSM) Thin Film Model Electrodes. J. Electrochem. Soc..

[56] Xiao G., Liu Q., Wang S., Komvokis V.G., Amiridis M.D., Heyden A., Ma S., Chen F. (2012). Synthesis and characterization of Mo-doped SrFeO_3−δ_ as cathode materials for solid oxide fuel cells. J. Power Sources.

[57] Liu B., Zhang Y., Zhang L. (2009). Oxygen reduction mechanism at Ba_0.5_Sr_0.5_Co_0.8_Fe_0.2_O_3−δ_ cathode for solid oxide fuel cell. Int. J. Hydrog. Energy.

[58] Escudero M.J. (2007). A kinetic study of oxygen reduction reaction on La_2_NiO_4_ cathodes by means of impedance spectroscopy. J. Electroanal. Chem..

[59] Marrero-López D., Dos Santos-Gómez L., Canales-Vázquez J., Martín F., Ramos-Barrado J.R. (2014). Stability and performance of nanostructured La_0.8_Sr_0.2_MnO_3_ cathodes deposited by spray-pyrolysis. Electrochim. Acta.

[60] Wang Y., Zhang L., Chen F., Xia C. (2012). Effects of doped ceria conductivity on the performance of La_0.6_Sr_0.4_Co_0.2_Fe_0.8_O_3−δ_ cathode for solid oxide fuel cell. Int. J. Hydrog. Energy.

[61] Yan L., Kavaipatti B., Chang K.-C., You H., Salvador P. (2011). Microstructural Effects on the Oxygen Exchange Kinetics of La_0.7_Sr_0.3_MnO_3_ Thin Films. ECS Meet. Abstr..

